# Necrosis of the Lower Jaw from Undeveloped Teeth

**Published:** 1870-11

**Authors:** J. H. Posley


### SELECTED ARTICLES.

---

### ARTICLE IV.

### *Necrosis of the Lower Jaw from Undeveloped Teeth.*

By J. H. Posley, Jr., M. D.

Mrs. B. æt. 30. Has had three children; health has been good until within the last year or two, when she became much debilitated from quickly succeeding pregnancies, and the care of a sick child, which died after a very protracted illness.

Her second dentition was irregular, two of the incisors of the lower jaw not making their appearance, and those which did come through being so uneven and misplaced that they were removed and false teeth substituted. About a year ago she noticed that the plate of her artificial teeth began to give her pain and uneasiness; soon after this an indolent swelling made its appearance upon her chin, just beneath the lower margin of the jaw; this was opened, but very little matter came from it; the opening did not heal up, but remained open with everted mucous looking edges; subsequently a similar swelling made its appearance about an inch distant from it on the other side of the symphysis menti, and was also opened; like the first it gave exit to very little matter, and did not heal up, presenting in a short time an appearance exactly similar to the first.

When I first saw her (June 1869), these two sinuses were still open and discharging, presenting very markedly the peculiar appearance of openings leading to diseased bone; upon introducing a probe into either of them it came into contact with exposed bone. There was considerable inflammatory thickening around them; the integument was of a dark red or purplish color, and there was considerable elongation and projection of the chin.

Owing to the irritation produced by the examination, an abscess formed between the openings, which in contradistinction to the former ones, was painful, hot and throbbing; was opened in a few days, and gave exit to a considerable quantity of healthy well formed pus, but did not heal up, remaining fistulous like the others; there were now three openings along the margin of the jaw, one about the symphysis, and one about half an inch distant on either side of it.

For reasons which made delay advisable, nothing was done until Nov. 23d, 1868, when an operation was performed for the removal of the dead bone.

An incision was made along the lower margin of the bone, as far back as possible, extending about an inch and a half on each side of the symphysis and through the middle of the open sinuses, and the bone exposed. No detached bone was found, but that portion forming the projection of the chin was rough and carious; the whole of the diseased portion was removed by the saw, aided by bone nippers and chisel, it being impossible to complete the section with the saw without enlarging the external wound, which it was desirable to avoid if possible.

On the surface of the remaining bone was found a small cavity, lying loosely in which was an incisor tooth, fang downwards, the crown and neck of which were carious. It was removed and the cavity scooped out; it communicated with a small circular opening in the portion of bone removed, which in its turn led to one of the openings in the soft parts; on inspecting the portion of bone removed, the section was found to have passed through another tooth, an incisor apparently not perfectly developed, but quite sound and healthy; it was firmly imbedded in the bone, but partially surrounding it, and leading to another circular opening communicating with the other sinus in the soft parts, could be seen an irregular groove, as though nature were engaged in undermining it and effecting its detachment. The surface bone remaining seemed to be perfectly healthy. The patient made a good recovery, the wound healing up slowly but soundly, the cicatrix retracted and out of sight, and the red inflamed prominence of skin quite gone.

I feel compelled to regard this case as unusual and therefore interesting, as I cannot find any similar one referred to in Heath's very complete and recent monograph on diseases of the jaws, which I have looked over for that purpose. In Forgeret on Dental Anomalies, is an account of errant teeth in cysts of the jaw. It may perhaps be a fair question whether these decayed teeth were the exciting cause of the bone inflammation, or whether the inflammation arising spontaneously, or otherwise excited, was identified and localized around these foreign bodies. The main reason against the first supposition is this having remained so long without giving rise to any trouble, but it must be noted that this lady's feeble and cachetic state of health at this time may have determined an attack of inflammation from a cause which would not otherwise have given rise to it.—*Medical Gazette.*

